# Therapeutic Cell Repopulation of the Liver: From Fetal Rat Cells to Synthetic Human Tissues

**DOI:** 10.3390/cells12040529

**Published:** 2023-02-06

**Authors:** David A. Shafritz, Mo R. Ebrahimkhani, Michael Oertel

**Affiliations:** 1 Department of Medicine, Albert Einstein College of Medicine, Bronx, NY 10461, USA; 2 Division of Experimental Pathology, Department of Pathology, University of Pittsburgh, Pittsburgh, PA 15213, USA; 3 Pittsburgh Liver Research Center (PLRC), University of Pittsburgh, Pittsburgh, PA 15213, USA; 4 McGowan Institute for Regenerative Medicine, University of Pittsburgh, Pittsburgh, PA 15219, USA

**Keywords:** fetal liver stem/progenitor cells, iPS cells, cell transplantation, Dlk-1, human organoids

## Abstract

Progenitor cells isolated from the fetal liver can provide a unique cell source to generate new healthy tissue mass. Almost 20 years ago, it was demonstrated that rat fetal liver cells repopulate the normal host liver environment via a mechanism akin to cell competition. Activin A, which is produced by hepatocytes, was identified as an important player during cell competition. Because of reduced activin receptor expression, highly proliferative fetal liver stem/progenitor cells are resistant to activin A and therefore exhibit a growth advantage compared to hepatocytes. As a result, transplanted fetal liver cells are capable of repopulating normal livers. Important for cell-based therapies, hepatic stem/progenitor cells containing repopulation potential can be separated from fetal hematopoietic cells using the cell surface marker δ-like 1 (Dlk-1). In livers with advanced fibrosis, fetal epithelial stem/progenitor cells differentiate into functional hepatic cells and out-compete injured endogenous hepatocytes, which cause anti-fibrotic effects. Although fetal liver cells efficiently repopulate the liver, they will likely not be used for human cell transplantation. Thus, utilizing the underlying mechanism of repopulation and developed methods to produce similar growth-advantaged cells in vitro, e.g., human induced pluripotent stem cells (iPSCs), this approach has great potential for developing novel cell-based therapies in patients with liver disease. The present review gives a brief overview of the classic cell transplantation models and various cell sources studied as donor cell candidates. The advantages of fetal liver-derived stem/progenitor cells are discussed, as well as the mechanism of liver repopulation. Moreover, this article reviews the potential of in vitro developed synthetic human fetal livers from iPSCs and their therapeutic benefits.

## 1. Introduction

The liver regulates many essential physiological processes that require the maintenance of a constant liver mass. However, chronic injuries disrupt the hepatostat, resulting in diminished regenerative capacity and impaired hepatic function [1]. Chronic liver diseases (CLDs) lead to cirrhosis and cancer, major causes of death [2]. Organ transplantation is presently the only therapeutic option, but organ shortage is a fundamental limitation [3,4]. Thus, new therapeutic approaches are in high demand, in turn requiring better comprehension of essential mechanisms involved in the progression of chronic diseases. Decades ago, hepatocyte infusion became a promising alternative to liver transplantation [5]. Landmark studies in rodents have shown that hepatocytes repopulate the liver, but only under very specialized experimental conditions [6,7,8,9]. Importantly, hepatocytes do not significantly replace tissue mass in normal livers [10], and therefore, studies have been focused on identifying other cell sources that efficiently repopulate the liver. To date, rat fetal liver stem/progenitor cells (FLSPCs) are the only cells that can significantly repopulate a normal liver and replace functional tissue mass [11,12]. Moreover, different protocols have been developed to produce engineered human induced pluripotent stem cells (hiPSCs). Studies are currently aiming to generate human hepatic cell lines with a growth advantage in vitro that can be used for novel cell-based therapies in patients with liver disease.

## 2. Cell Transplantation Models and Donor Cell Candidates

### 2.1. Major Cell Transplantation Models

Decades ago, elegant studies demonstrated a “Proof-of-principle” that transplanted rodent hepatocytes significantly replace functional liver mass and exhibit great translational potential. Rhim et al. [6] infused mature lacZ-positive hepatocytes into transgenic mice, which over-express an *albumin-urokinase plasminogen activator (uPA)* fusion construct, a hepatotoxic transgene leading to severe liver injury [13] Using X-gal staining to detect lacZ donor cells, the authors observed up to 80% tissue replacement after cell infusion [6]. Two years later, in 1996, Grompe’s team [7] described a murine model of hereditary tyrosinemia type 1, characterized by fumarylacetoacetate hydrolase deficiency (*Fah^−/−^*), resulting in lethal liver dysfunction. Transplanted hepatocytes repopulated more than 90% of the *Fah^−/−^* mouse liver within two months and restored functional tissue mass [7]. Both cell transplantation models demonstrated that genetic modifications of the recipient liver create hepatic microenvironments, leading to a strong growth advantage for donor-derived hepatocytes. Twenty years later, Hui’s team [14] generated a *Fah*^−/−^ rat model, which harbored the major characteristics of human hereditary tyrosinemia type 1 as well as developed advanced liver fibrosis, which have not been seen in *Fah^−/^*^−^ mice [7] and *Fah^−/−^* swine [15]. In *Fah^−/−^* rats, the authors showed efficient repopulation of hepatocytes, which prevented fibrosis progression [14].

In 1998 and 1999, two research groups at Einstein described cell transplantation models, in which the authors blocked hepatocyte proliferation and accomplished long-lasting cell cycle arrest in rat livers by pretreating the animals with retrorsine [8] or irradiation of the recipient liver [9] in conjunction with two-thirds partial hepatectomy prior to cell infusion. Both treatments led to near-total liver tissue replacement by transplanted hepatocytes after several months. A few years later, Petersen’s team [16] used the retrorsine derivative monocrotaline as an alternative to retrorsine-based hepatocyte transplantation in mice and rats to create an effective selective pressure for donor hepatocytes.

The described landmark studies not only demonstrate the ability of rodent hepatocytes in effectively regenerating damaged liver tissue mass, but they also enable the evaluation of the repopulation potential of alternative cell sources to hepatocytes (*see below*). In addition, several immunocompromised rodent models were developed that can be used to study the expansion properties of human-derived/engineered cells, e.g., *uPA^tg(+/−)^*/*Rag-2^−/−^*, *uPA^tg(+/−)^*/SCID, *uPA^tg(+/−)^*/*Rag-2^−/−^*/*γc^−/−^*, *Fah^−/−^*/*Rag-2^−/−^*/*Il-2rg^−/−^*/NOD (FRGN), *Fah^−/−^*/*Rag-2^−/−^*/*Il-2Rγc^−/−^*/SCID (FRGS), TK-NOG mice and Sprague Dawley/*Rag-2^−/−^*/*Il-2rg^−/−^* (SRG) rats [17,18,19,20,21,22,23,24]. 

### 2.2. Various Cell Sources for Transplantation

The availability of “healthy” hepatocytes for human cell transplantation is limited, and therefore, research is focused on the evaluation of alternative cell types. Stem and progenitor cells represent a promising cell source of functional hepatocytes because they exhibit high proliferative activity and differentiate into hepatocytes and/or bile ducts. 

***Oval cells*** [25] are adult liver progenitor cells that can be induced in rodent models by 2-acethyl amino-fluorene, a choline-deficient diet, or D-galactosamine [25,26,27]. Cell transplantation studies have shown that oval cells are capable of repopulating recipient mouse and rat livers, but only in hepatic microenvironments with induced strong growth advantage for transplanted cells, i.e., under *selective conditions*, such as in *Fah*^−/−^, monocrotaline-treated or retrorsine-treated liver [28,29,30].

In 1999, Petersen et al. [31] reported that ***bone marrow cells*** form oval cells when transplanted into injured rat livers. Following studies showed evidence that bone marrow cells can differentiate into hepatocytes [32], and up to 50% repopulation was achieved under highly selective conditions in the *Fah*^−/−^ mouse [33]. However, the obtained tissue replacement after bone marrow cell infusion occurred through cell fusion between host hepatocytes and donor-derived myelomonocytic cells, rather than through cell differentiation [34,35,36]. Additional studies determined that transplanted bone marrow cells did not give rise to increasing oval cells in injured livers [37].

Human ***umbilical cord blood***-derived hematopoietic stem cells, another candidate for cell transplantation, have shown engraftment and hepatocytic differentiation potential after infusion into nonobese diabetic/severe combined immunodeficient (NOD/SCID) mice. However, therapeutic repopulation levels have not been observed [38,39,40]. Other research has focused on murine and human ***mesenchymal stem cells (MSCs)***, which were isolated from bone marrow or umbilical cord blood. Several reports have demonstrated that transplanted non- or pre-conditioned MSCs are capable of engrafting and differentiating into hepatocyte-like cells in immunodeficient NOD/SCID, *Pfp/Rag-2^−/−^* or SCID mice [41,42,43,44,45]. Christ’s team transplanted pre-differentiated rat and human adipose tissue-derived MSCc into retrorsine-treated dipeptidyl-peptidase IV-negative (DPPIV^−^) F344 rats or *Pfp/Rag-2^−/−^* mice and observed large hepatocyte-like cell clusters at 10 weeks after cell infusion [46,47]. Although these promising studies identified another donor cell source, significant tissue replacement was not achieved in most of these studies. Nevertheless, MSC transplantation has received increasing attention over the years because of its therapeutic effects based on paracrine effects through the secretion of growth factors and cytokines, e.g., hepatocyte growth factor (HGF), epidermal growth factor (EGF), tumor necrosis factor alpha (TNF-a) and interleukin 6 (IL-6), which promote liver regeneration and the replacement of damaged hepatocytes [48,49]. In a recent review, Liu et al. [50] summarized 160 clinical trials in which stem cells were used for the treatment of end-stage liver diseases, cancer and fibrosis/cirrhosis. The majority of used donor cells were MSCs derived from umbilical cord, bone marrow and adipose tissue [50]. 

It was shown that ***amniotic epithelial cells (AECs)*** isolated from human term placenta have the potential to differentiate into all three germ layers, including tissues of endodermal origin (i.e., liver) [51]. After the transplantation of human or rat AECs into livers of retrorsine-treated SCID/beige mice or DPPIV^−^ F344 rats, respectively, cells were capable of engrafting and differentiating into hepatocytes. Although repopulation levels were very low with human AECs (up to 1%), rat-derived AECs formed big cell clusters containing up to 4000 cells at 12 months, representing a promising cell source for transplantation [52]. In subsequent studies, Strom’s research group demonstrated that human AEC transplantation significantly extended survival and normalized the body weight in a mouse intermediate maple syrup urine disease (iMSUD) model [53]. Other human AEC transplantation studies achieved reduced liver fibrosis in CCl_4_-treated immunocompetent C57BL/6 mice [54,55] or restored the glycosaminoglycan (GAG)-degrading enzyme α-l-iduronidase (IDUA) function in the livers of mucopolysaccharidosis type 1 (MPS1) NOD.129(B6)-*Prkdc^scid^ Idua^tm1Clk^* mice [56]. However, the achieved therapeutic benefits resulted in paracrine effects of AECs, but significant repopulation levels were not shown in these cell transplantation models.

In 1981, a groundbreaking study described the isolation of murine pluripotent ***embryonic stem cells (ESCs*** [57]. Two decades later, the differentiation potential of ESCs into hepatocytes was investigated [58], which was the basis for several subsequent studies evaluating the hepatic differentiation and repopulation potential of rodent and human ESCs. However, therapeutic repopulation levels were not observed, and ethical concerns and teratoma formation reduced interest in ESCs as a potential donor cell source for human cell transplantation [22,59,60,61].

Evidence that ***fetal liver cells*** differentiate into both hepatic lineages was first shown by Leduc and Wilson in 1963 [62] and Ebata and Mito in 1985 [63]. The authors transplanted small tissue fragments isolated on embryonic days (EDs) 13, 14 and/or 18 into the spleens of mice or Wistar rats and observed not only hepatocyte differentiation and long-term survival up to 20 months in mice, but also differentiation into bile duct cells and hepatocytic nodule formation at 12 months in rats. Almost 20 years later, studies have described several methods for the isolation and purification of murine ED13.5 or 14.5 fetal liver cells with hepatic differentiation potential, using fluorescence-activated or magnetic cell sorting (FACS or MACS) [64,65,66]. After the transplantation of enriched epithelial ED12.5 cells into retrorsine/CCl_4_-treated DPPIV^−^ C57BL/6 mice, cells engrafted and up to 80% repopulation was observed at 4 months [67]. Kubota and Reid [68] isolated and enriched epithelial rat ED13 fetal liver cells that differentiated into both hepatic lineages. Moreover, a c-Met-specific antibody was used to purify rat ED14 fetal liver cells, which formed hepatocytic cell clusters after infusion into retrorsine-treated animals [69]. In contrast, under *non-selective conditions*, subsequent studies demonstrated that rat fetal liver cells can replace >20% liver mass at 6 months [12]. The advantages of rat fetal liver cells are discussed in *CHAPTER 3*.

Today, studies focus on generating human hepatic cell lines for cell transplantation, e.g., via using engineered human ***induced pluripotent stem cells (hiPSCs)*** [70,71,72,73] or via conversion from fibroblasts [73,74,75]. Using different protocols for generating hepatocyte-like cells from hiPSCs [73], induced hepatocyte-like cells (iHeps) exhibit some ability to repopulate rodent livers under selective conditions [74]. The potential of hiPSCs is discussed in *CHAPTER 4*.

## 3. Rat Fetal Liver Cell Transplantation

Rats, as an important model organism for biomedical research, have many advantages over other animal models [14]. One of the most frequently used cell transplantation models is the Fisher (F)344 rat [76], which utilizes the transplantation of DPPIV^+^ donor cells, isolated from wild-type F344 rats, infused into mutant DPPIV^−^ F344 rats [77]. In this syngeneic cell transplantation model, transplanted and repopulating donor cells can be detected with enzyme histochemistry or immunohistochemistry for DPPIV (CD26) in the host hepatic parenchyma. Using the F344 rat model, five major observations were made using rat FLSPCs. *First*, massive tissue replacement was achieved in the normal liver. *Second*, tissue replacement by transplanted cells occurred through a mechanism akin to cell competition. *Third*, activin A, a multifunctional cytokine produced in the liver parenchyma, played a key role during cell competition. *Fourth*, injured hepatic microenvironments with advanced fibrosis/cirrhosis could be effectively replaced by transplanted cells, and fibrosis could be reversed. *Fifth*, fetal liver-derived endodermal stem cells could also differentiate into non-hepatic lineages driven by the diseased host environment.

### 3.1. Repopulation by Hepatic Fetal Liver Stem/Progenitor Cells in a Normal Liver

In 2001, Shafritz’s team reported that rat epithelial ED14 fetal liver stem/progenitor cells (FLSPCs) repopulated ~7% of the recipient liver at 6 months after cell infusion in conjunction with two-thirds partial hepatectomy [11]. Subsequently, Oertel et al. [12] transplanted high numbers of unfractionated rat fetal liver-derived cells and observed >20% tissue replacement at 6 months.

The authors purified FLSPCs using magnetic bead cell sorting (MACS) for the cell surface marker δ-like 1 (Dlk-1) to 95% homogeneity that contained the gene expression characteristics typical for hepatic stem/progenitor cells, as well as all the repopulation potential of unpurified FLSPCs [78]. Dlk-1, a glycoprotein highly expressed in human and murine ED12.5 fetal liver cells [66,79], is also expressed in adult rat liver progenitor cells [80,81]. These enriched Dlk-1-positive cell isolates contained all a-fetoprotein (AFP)^+^ hepatoblasts found in epithelial fetal liver cell fractions, and were also positive for E-cadherin and 20% of the cells cytokeratin (CK)-19^+^ [78]. Importantly, only a few cells within the repopulating Dlk-1^+^ fraction expressed the hematopoietic stem cell marker Thy-1 [82], a surface antigen previously detected in developing rat and human fetal livers [83,84].

Additional FLSPC transplantation studies discovered increasing repopulation levels in aging hepatic environments driven by the reduced regenerative capacity of the host liver [85]. Importantly, FLSPCs maintained their full repopulation potential after long-term storage at −80 °C and thawing, followed by transplantation into normal recipient rats [86].

To date, significant repopulation under *non-selective conditions* was only reported using the described rat cell transplantation model. This raises the question of whether this is an intra-species-specific phenomenon. There is evidence that mouse FLSPCs might replace hepatic tissue mass under non-selective conditions [67]; however, murine cell transplantation reports demonstrating significant tissue replacement in normal livers are still non-existent. Although reports about human fetal liver cell transplantations are limited [87,88,89,90], the majority of liver patients have been transplanted with hepatocytes [5,91]. It is unlikely that liver cells isolated from human fetuses will be used routinely for human cell therapy, primarily because of ethical concerns. The small number of such cells obtained from a single aborted human fetus at mid-gestation (equivalent to rat fetal liver cells at ED13-14) is not sufficient for effective repopulation. In addition, the use of pooled cryopreserved liver cells from multiple aborted fetuses can also increase the tendency for rejection by the host. However, established rodent cell transplantation models have been an excellent experimental tool, building a framework to study and engineer efficient repopulation and the underlying mechanisms by which this occurs.

### 3.2. Cell Competition Drives Liver Repopulation

Years ago, it was demonstrated that ED14 rat fetal liver cells repopulate the normal host liver environment via a mechanism akin to cell competition [12], originally described in *Drosophila* wing development [92]. Increasing knowledge in cell competition has accumulated within the past two decades. In a recent article, Bowling et al. [93] reviewed cell competition as a striking process characterized by the elimination of less fit (loser) cells by more fit (winner) cells, a process characterized by three steps. *First*, cell competition occurs between two different fit cells in the tissue. *Second*, more fit cells eliminate less fit cells via different mechanisms, and *third*, tissue replacement occurs while sustaining constant tissue mass. 

**Figure 1:**
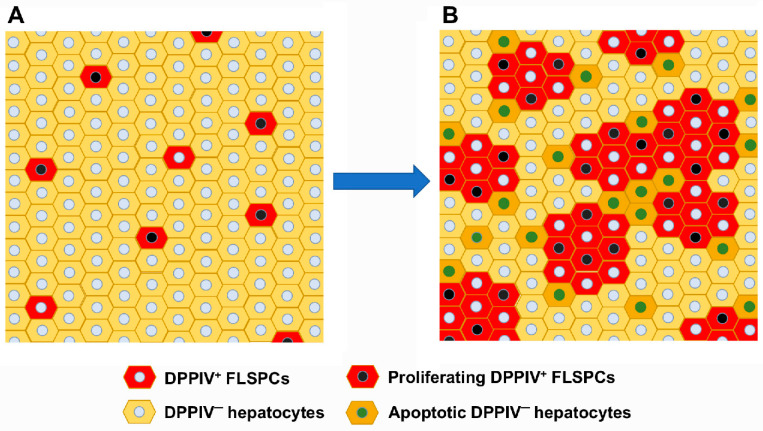
Repopulation via cell competition by transplanted DPPIV^+^ FLSPCs into DPPIV^−^ host liver: (**A**) Engrafted, highly proliferative fetal liver cells. (**B**) Dividing FLSPCs form cell clusters and induce apoptosis in host parenchyma cells.

**Figure 2:**
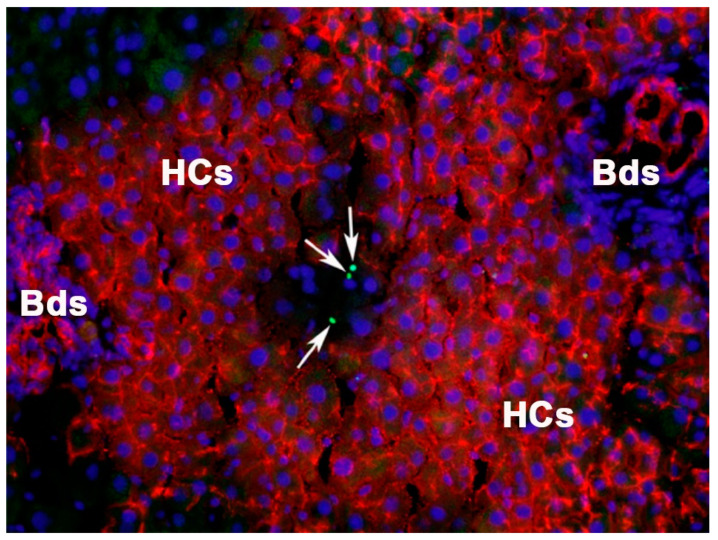
Apoptotic bodies in DPPIV^−^ host hepatocytes being eliminated in a large coalescing cluster of transplanted DPPIV^+^ FLSPCs. Simultaneous detection of transplanted (immunohistochemistry for DPPIV; *red*) and apoptotic cells (TUNEL assay; *green*, highlighted by arrows). 4′,6-diamidino-2-phenylindole (DAPI) was used for staining of nuclei (*blue*). HCs: hepatocytes; Bds: bile ducts.

Cell transplantation studies, in conjunction with two-thirds partial hepatectomy, which is required for cell engraftment into a normal liver, showed that rat FLSPCs, with their high proliferative activity, generate new tissue mass in host parenchyma exhibiting lower proliferation rates. They act as winners by cell-competition-induced apoptosis to make space in the liver by inducing host apoptosis near the boundaries of the transplanted cell clusters in order to maintain the original liver size (Figure 1 and Figure 2) [12], a phenomenon also reported in *Drosophila* [94,95]. To date, certain mechanisms have been discussed through which winners out-compete loser cells [93,96,97]. However, the exact mechanism(s) of how growth-advantaged fetal liver cells eliminate growth-disadvantaged host hepatocytes remains not fully understood. The observation of increasing repopulation levels by FLSPCs in aging host livers, characterized by increasing activin A levels and its target gene *p15^INK4b^*, suggested activin A/p15^INK4b^ signaling as the pathway driving cell competition [85]. Because FLSPCs are resistant to the growth-inhibitory effects of activin A due to reduced activin A receptor expression [85], endogenous host hepatocytes—the main producers of activin A [98], which is a well-known hepatocyte growth inhibitor [98,99,100,101]—become losers. However, it is unlikely that the proliferation of FLSPCs alone drives cell competition. There is evidence that metabolic activity impacts cell fitness [93]. Activin A down-regulates many genes involved in hepatocyte metabolism [85,98], another piece of evidence suggesting that activin A signaling is involved in liver tissue replacement by making FLSPCs superior over host hepatocytes, resulting in tissue clearance of the later.

### 3.3. Replacement of Functional Tissue Mass in Diseased Livers

The four major cell transplantation studies [6,7,8,9] described in Section 2.1 demonstrate the capability of hepatocytes to effectively regenerate damaged hepatic tissue mass; however, these models do not represent common clinical circumstances. To study the influence of the diseased microenvironment on the outcome of cell transplantation, established rodent disease models must be used. Using two fibrosis models (fibrosis induced by thioacetamide [TAA], biliary fibrosis by bile duct ligation [BDL]) in DPPIV^−^ F344 rats, dramatic changes in activin A/p15^INK4b^ and its target genes were detected in fibrotic livers (*Oertel, unpublished data*). Yovchev et al. [102] transplanted unfractionated ED15 FLSPCs into rat livers with TAA-induced advanced fibrosis/cirrhosis. After FLSPC infusion into the portal vein, small cell clusters were already detected at day 7, and repopulation levels with up to 41% tissue replacement were observed at 4 months. Importantly, even after transplantation without partial hepatectomy, FLSPCs engrafted and differentiated into both hepatic lineages, hepatocytes and bile duct epithelial cells, and >25% repopulation was achieved, associated with reduced fibrosis [102]. These observations suggest that changes in the activin A/p15^INK4b^ axis in fibrotic livers create local tissue regions with impaired regeneration, which enables engraftment and drives the compensatory proliferation of infused cells. Using a second fibrosis model, FLSPCs migrated and engrafted in the fibrotic liver and formed hepatic cell clusters expressing hepatocyte nuclear factor (HNF)4a and claudin (Cldn)-7 at 2 months after cell infusion into the spleens of bile-duct-ligated rats without partial hepatectomy [103]. A substantial number of cells that engrafted in the spleen differentiated into hepatocytes and bile duct structures. Moreover, FLSPCs differentiated into non-hepatic endodermal lineages expressing caudal type homeobox 2 [Cdx2], pancreatic and duodenal homeobox 1 (Pdx1) and keratin 13 (CK-13). Therefore, FLSPCs contain multipotent endodermal stem cells that colonize the diseased splenic microenvironment and differentiate into multiple gastrointestinal tissues, including that of the liver, pancreas, intestine, and esophagus [103].

The influence of a diseased environment on the outcome of cell transplantation was also demonstrated in studies using mature hepatocytes transplanted into bile-duct-ligated rats [104]. Ongoing biliary fibrosis forces the selective growth advantage and phenotype transition of ectopic-infused hepatocytes. After one week, engrafted hepatocytes showed biliary epithelial marker expression (SRY-related high-mobility group box [Sox]-9), and after a second week, clear hepatocyte-derived ductules were observed. At two months, ten % of the transplanted hepatocyte-derived cell clusters contained bile duct structures— phenotype transdifferentiation [105,106] is driven by the secreted pleiotropic cytokine osteopontin [107] in fibrotic livers [104]. 

## 4. Human iPSC-Derived Cells and Application for Human Liver Diseases

### 4.1. Human iPSC-Derived Hepatocytes, Challenges and Opportunities

iPSC-derived cells are on their way to clinics (first-in-human clinical trial of iPSC-derived neural stem/progenitor cells in spinal cord injuries is a recent example) [108]. Additionally, cellular immunotherapies for cancer, such as lymphoma, have used off-the-shelf natural killer cell products derived from an induced pluripotent stem cell (iPSC) line [109]. In 2020, Japanese doctors transplanted hepatocytes derived from embryonic stem cells into a newborn baby suffering from a urea cycle disorder. This treatment decreased ammonium levels and provided a bridge for the recipient to eventually reach appropriate conditions for a liver transplant [110].

Induced hepatocytes, or iHeps, are developed either as a single cell population from iPSCs or within multicellular systems, such as organoids. iHeps have the potential to provide a cell source that is autologous and functionally amenable for therapeutic repopulation (reviewed extensively in refs. [73,111,112]). However, there are existing challenges with the generation of these cells and their performance in vivo: (**1**) Developed cells have low proliferative activity and often show aberrant signatures from other endodermal organs, which limits the engraftment, repopulation, and robustness of their fates in vivo. (**2**) There is a limited ability to control cell fate and long-term stability of iHeps after cell infusion. Specifically, using soluble factors to generate these cells limits the ability to control their fate of these cells after transplantation. (**3**) Signals from a physiological “human” niche, such as endothelial cells or pericytes, are widely missing (particularly after cell transplantation). (**4**) For final human applications, the scale of the cells and manufacturing enough of them for human-based therapeutics is a challenge and will be an issue considering their low proliferating capacity and the possible lack of appropriate niche signals.

Ongoing research is focused on advancing the maturation of cells; however, improved maturation may come at the cost of limiting the proliferative capacity of these cells. In vitro cultures may also promote the generation of aberrant signatures. Importantly, current culture media lack the physiological levels of factors necessary to capture the in vivo environment. Further work to identify the aberrant signatures of developed cells and to compare them with in vivo counterparts to direct the improvement of cell identity is critical for developing better therapeutic cells. Different computational methods have been developed to address this challenge [113,114]. Additionally, it has been shown that engineering gene regulatory networks in hepatic organoids enables the guided maturation of cells, which has also been accompanied by a decrease in aberrant signatures [115]. It was also shown that cells further undergo maturation after implantation [116]; therefore, developing cells with high proliferative capacity, such as fetal liver stem/progenitor cells, which can out-compete diseased cells or host hepatocytes, are an important objective to achieve maturation following implantation. However, an immature phenotype with an aberrant signature of other endodermal organs may result in maturation toward non-hepatocyte identities, as seen with fetal liver cells in a diseased microenvironment [103]. This clearly limits the safety profile of these cells.

### 4.2. Multilineage Human Fetal Liver Organoids and Their Therapeutic Benefits

Although human fetal livers can serve as a source of endodermal and mesoderm cells for regenerative medicine [117], access to fetal liver tissue is limited due to both ethical and practical reasons, as mentioned above. Therefore, the development of alternative cell sources with fetal liver-like characteristics is of tremendous value.

Recently, a human fetal liver organoid was developed that captures the complexity of the natural liver, including cell types, signaling cues and hematopoietic niche [115,118]. This platform was produced from hiPSCs via transiently expressing the *GATA6* transcription factor to trigger both endoderm and mesoderm differentiations and the co-differentiation of progenitor cells through reciprocal cell–cell interactions. Without further supplementation of growth factors, these cells co-differentiated in a stepwise fashion into a complex, fetal liver-like tissue relying on self-produced, more physiological concentrations of the signaling cues. Within two weeks, a vascularized hepatic-like tissue that contained CEBPa, AAT^+^ hepatoblasts, CD34^+^ endothelium and desmin^+^ stellate-like cells was generated. The developed tissue also contained DLK-1^+^ hepatoblast-like cells and showed the production of cell types that are normally found in developing fetal livers, such as nestin^+^ pericytes [115,118].

**Figure 3:**
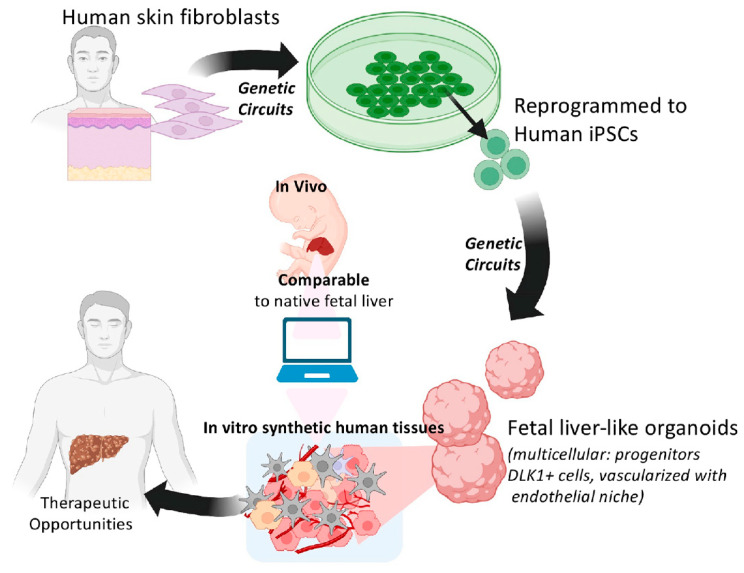
Generation of synthetic fetal liver organoids using human iPSCs can provide a novel platform for human liver repopulation during diseased conditions. Human skin cells can be reprogrammed to human iPSCs using reprogramming factors. Developed iPSCs can differentiate toward synthetic multicellular fetal liver organoids using, e.g., *GATA6* genetic circuits. Produced organoids can represent a human cell source with a potentially competitive advantage for therapeutic liver repopulation.

In contrast to primary cells, human iPSC fulfills a cell source that can be induced to differentiate, tested in vivo, assessed, and redesigned for better performance in regenerative therapies. With the advent of synthetic developmental biology, it will be possible to generate designer cells that mimic the key specifics of in vivo tissues [115,119]. For example, synthetic fetal liver tissue may provide key fetal cell courses necessary for therapeutic repopulation. Additionally, via genome editing technologies [119], one can re-design certain characteristics for the generation of cells with more potent repopulation capacity and safety switches for controlled in vivo monitoring and functions (Figure 3). Hence, combining iPSCs and synthetic biology with knowledge obtained from fetal liver cells will enable more effective therapeutic liver repopulation. 

## 5. Concluding Remarks

Cell transplantation with an emphasis on candidate cells represents a promising alternative to whole liver transplantation [120,121,122,123,124]. In this regard, recent research has focused on the mechanisms through which growth-advantaged cells out-compete host liver cells, as well as on the characteristics of liver microenvironments that augment tissue replacement by transplanted cells. Cell competition, or ‘survival of the fittest cells’, is a universal process involved in the regulation of organ size, the elimination of mutant and injured cells, aging, as well as cancer [93,125]. Besides FLSPCs, hepatocyte transplantation studies have also shown evidence that cell competition is involved in tissue repopulation. Pasciu et al. [126] reported that transplanted young rat hepatocytes, which normally do not repopulate a normal liver [77], formed cell clusters in aged host liver. Hepatocytes transduced ex vivo with a lentivirus vector encoding the human YapERT2 fusion protein exhibited a growth advantage, driving repopulation in normal rat livers, and 10% and 14% tissue replacement was achieved at 6 and 12 months, respectively [127,128], representing repopulation levels with a therapeutic effect in human patients [129]. Despite all these amazing findings, molecular pathways involved in cell competition driving liver repopulation still require further study. 

Using experimental models similar to human fibrosis/cirrhosis, studies have demonstrated that transplanted FLSPCs generate new tissue mass in cirrhotic livers, reversing fibrosis. These studies overturned the long-held belief that cirrhotic microenvironments are contraindications for human cell therapies. Moreover, fetal livers harbor endodermal stem cells that can form multiple gastrointestinal tissues, as well as candidate cells with therapeutic potential for diseased organs beyond the liver. Such observations are important contributions to the field of regenerative medicine and build the framework to generate designer cells derived from iPSCs with favorable fetal liver stem/progenitor cell features in the future.
